# Impact of Pre-Transplant Frailty on Early Outcomes Following Liver Transplantation: A Propensity-Matched Multicenter Cohort Study

**DOI:** 10.3390/jcm15114003

**Published:** 2026-05-22

**Authors:** Noor Albusta, Mohamed Abdulla, Sara Isa, Hussain Alrahma

**Affiliations:** 1 Department of Internal Medicine, Lahey Hospital and Medical Center, Burlington, MA 01805, USA; 2 Department of Medicine, Royal College of Surgeons in Ireland, Medical University of Bahrain, Adliya P.O. Box 15503, Bahrain; 16123638@rcsi.com (M.A.); 19201227@rcsi.com (S.I.); 3 Department of Gastroenterology and Hepatology, Salmaniya Medical Complex, Manama P.O. Box 12, Bahrain; hrahma1@hospitals.gov.bh

**Keywords:** liver transplantation, frailty, sarcopenia, acute kidney injury, mortality, propensity score matching, mechanical ventilation, prehabilitation, outcomes

## Abstract

**Background/Objectives:** Frailty is a validated predictor of waitlist mortality and perioperative risk in liver transplant candidates, but its association with early post-transplant outcomes in large real-world cohorts remains incompletely characterized. This study evaluated the association between administratively defined pre-transplant frailty and early clinical outcomes following liver transplantation. **Methods:** We conducted a retrospective cohort study using the TriNetX US Collaborative Research Network. Adults undergoing first-time isolated liver transplantation through February 2026 were included. Frailty was identified using ICD-10-CM codes for frailty, sarcopenia, cachexia, weakness, abnormal gait/mobility, or reduced mobility documented within 12 months before transplantation; patients coded only for nonspecific weakness were excluded from the frailty cohort. Patients underwent 1:1 propensity score matching using 18 baseline covariates, including demographics, comorbidities, laboratory values, albumin, and MELD-Na. The primary outcome was all-cause mortality at 7, 30, and 90 days. Secondary outcomes included acute kidney injury, prolonged mechanical ventilation, vasopressor requirement/hemodynamic instability, renal replacement therapy, ICU and hospital length of stay, and 90-day readmission. Sensitivity analyses used a restrictive ≥ 2-code frailty definition and substituted MELD 3.0 for MELD-Na in the propensity model. **Results:** Among 4860 eligible recipients, 742 had administratively defined frailty and 4118 did not. After matching, 730 patients remained in each group with well-balanced covariates. Administratively defined frailty was associated with higher mortality at 7, 30, and 90 days, with numerically smaller relative risks at later time points. It was also associated with higher risks of acute kidney injury, prolonged mechanical ventilation, vasopressor requirement/hemodynamic instability, renal replacement therapy, longer ICU and hospital stays, and 90-day readmission. Findings were directionally consistent in both sensitivity analyses. Etiology-stratified analyses were exploratory and showed no statistically significant heterogeneity across liver disease etiologies. **Conclusions:** In this large propensity-matched multicenter cohort, administratively defined pre-transplant frailty was associated with worse early outcomes after liver transplantation. Because frailty and several outcomes were identified using structured EHR and administrative data, findings should be interpreted as associative and hypothesis-generating. Prospective studies using validated frailty instruments and granular donor, intraoperative, and center-level variables are needed to confirm these findings.

## 1. Introduction

Frailty is a multidimensional syndrome of diminished physiologic reserve that confers vulnerability to adverse outcomes following physiologic stressors [1,2]. In patients with cirrhosis and end-stage liver disease, frailty has been consistently associated with waitlist mortality, hospitalization, and impaired functional recovery [1,2]. The Liver Frailty Index (LFI), a validated performance-based tool incorporating grip strength, chair stands, and balance, has emerged as the reference standard for frailty assessment in this population and is endorsed by the 2025 AASLD/AST Practice Guideline on Liver Transplantation for pre-transplant evaluation [2,3,4].

The landmark multicenter FrAILT study (*n* = 1166 recipients across 8 US centers) demonstrated that pre-transplant frailty (LFI ≥ 4.5) was independently associated with post-transplant mortality (adjusted HR 2.13; 95% CI: 1.39–3.26), prolonged hospitalization (OR 2.00), prolonged ICU stay (OR 1.56), and nonhome discharge (OR 2.50) [5]. More recently, the Liver Transplant Comorbidity Index (LTCI) integrated frailty with coronary artery disease, hepatocellular carcinoma, renal dysfunction, and diabetes to predict 3-year post-transplant mortality, further establishing frailty as a central component of composite risk stratification [6]. Importantly, liver transplantation has been shown to provide net survival benefit at all levels of frailty, underscoring that the clinical goal is not to exclude frail candidates but to optimize perioperative care and risk stratification [4,7].

Despite these advances, several knowledge gaps persist. First, the FrAILT study evaluated frailty using the Liver Frailty Index but did not report granular early post-operative outcomes such as AKI, RRT, vasopressor requirement, or prolonged mechanical ventilation [5]. Second, the temporal dynamics of administratively defined pre-transplant frailty-associated risk—whether associations are strongest immediately postoperatively and numerically attenuate over time—have not been systematically characterized. Third, whether administratively defined pre-transplant frailty-outcome associations differ by underlying liver disease etiology remains unexplored. Fourth, emerging evidence on prehabilitation programs suggests that frailty may be modifiable before transplantation, with demonstrated improvements in LFI and 6 min walk test performance, but identifying which early outcomes are most associated with frailty is essential to guide prehabilitation targets [8,9].

To address these gaps, we conducted a large multicenter retrospective cohort study using the TriNetX US Collaborative Research Network [10,11]. This study aimed to evaluate the association between administratively defined pre-transplant frailty and a comprehensive spectrum of early post-transplant outcomes, describe temporal patterns in these associations, and explore etiology-specific differences in frailty-outcome associations.

## 2. Methods

### 2.1. Data Source and Study Design

We performed a retrospective cohort study using the TriNetX US Collaborative Research Network, a federated real-world electronic health record database and analytics platform provided by TriNetX, LLC (Cambridge, MA, USA). Available data include demographics, diagnoses (ICD-10-CM), procedures (CPT/ICD-10-PCS), medications, laboratory values, and vital signs. This study did not require institutional review board approval because the data were de-identified and HIPAA-compliant. The study was conducted in accordance with the STROBE guidelines for observational research.

Because TriNetX is a federated EHR network, patient-level chart review, detailed donor information, intraoperative variables, ventilator flow-sheet data, urine output, and center-specific transplant practices are not uniformly available. Therefore, exposures and outcomes were defined using structured diagnosis, procedure, medication, laboratory, and encounter data.

### 2.2. Study Population and Cohort Definitions

We identified all adult patients (≥18 years) who underwent first-time, isolated liver transplantation using ICD-10-PCS codes (0FY00Z0, 0FY00Z1, 0FY00Z2) and CPT code 47135. The index date was defined as the date of the transplant procedure.

Patients were classified into two cohorts based on the presence or absence of documented pre-transplant frailty within 12 months before the index transplant date:

Frailty cohort: Patients with at least one of the following ICD-10-CM codes docu-mented within 12 months before transplantation: R54 (age-associated physical debili-ty/frailty), M62.84 (sarcopenia), M62.81 (muscle weakness, generalized), R64 (cachexia), R63.4 (abnormal weight loss), R26.89 (other abnormalities of gait and mobility), Z74.09 (other reduced mobility), or R53.1 (weakness). To improve specificity, patients coded only with R53.1 (weakness) without any other qualifying code were excluded from the frailty cohort. This coding strategy was intended to identify an administratively defined, claims-based frailty phenotype rather than directly measured physiologic frailty. A single qualifying frailty-related ICD-10-CM code within 12 months before transplantation was sufficient for primary cohort assignment. This approach differs from performance-based frailty instruments such as the Liver Frailty Index, which directly assesses grip strength, chair stands, and balance.

Non-frailty cohort: Patients without any of the above codes documented within 12 months before transplantation.

Exclusion criteria (applied uniformly to both cohorts): age <18 years; incomplete demographic data (missing age, sex, or race); multiorgan transplantation (simultaneous liver-kidney, liver-heart, or liver-lung); retransplantation; acute/subacute hepatic failure due to trauma, acetaminophen overdose, or other acute toxic etiologies (ICD-10-CM K71.1x, T39.1x); and insufficient baseline data for propensity matching (defined as missing ≥3 of the 18 matching covariates).

### 2.3. Baseline Characteristics and Covariates

Eighteen baseline covariates were collected for propensity score matching and multivariable adjustment, including demographic variables (age, sex, and race/ethnicity [White, Black, Hispanic]); comorbidities documented within 12 months before transplant (type 2 diabetes mellitus [E11.x], hypertension [I10–I16], chronic kidney disease [N18.x], hepatic encephalopathy [K72.x, G93.4x], ascites [R18.x], hepatocellular carcinoma [C22.0], and pre-transplant dialysis [Z99.2]); and laboratory values measured within 90 days before transplant (total bilirubin, INR, creatinine, sodium, and albumin). MELD-Na was also included as a composite severity variable and was calculated from bilirubin, INR, creatinine, and sodium using the standard OPTN formula. MELD-Na was retained as the primary composite marker of liver disease severity because it remains widely used in transplant outcomes literature and allows direct comparison with prior landmark studies, including the FrAILT cohort and the Liver Transplant Comorbidity Index [5,6].

### 2.4. Study Endpoints

Primary outcome: All-cause mortality at 7, 30, and 90 days after liver transplantation.

Secondary outcomes:

- Acute kidney injury (AKI): Acute kidney injury (AKI) was defined using ICD-10-CM N17.x codes within the specified postoperative follow-up window. KDIGO-based AKI staging was not used because granular urine output data and sufficiently time-stamped serial creatinine values are not uniformly available across all participating healthcare organizations in the TriNetX federated platform [12]. Therefore, this definition likely captures clinically recognized AKI, particularly moderate-to-severe events, rather than all creatinine-defined AKI episodes.
- Prolonged mechanical ventilation: Prolonged mechanical ventilation was defined as >48 h of continuous mechanical ventilation using ICD-10-PCS code 5A1955Z or CPT codes 94002/94003 with duration >48 h. Ventilator flow-sheet data, ventilator settings, and exact extubation timing were not uniformly available in TriNetX; therefore, procedure-based definitions were used to ensure reproducibility across participating healthcare organizations.
- Vasopressor requirement or hemodynamic instability: This was defined by administration of norepinephrine, vasopressin, epinephrine, or phenylephrine, or ICD-10-CM coding for shock (R57.x), within the follow-up window. Because vasopressor dose, duration, and indication were not uniformly available, this outcome was intended to capture clinically significant postoperative hemodynamic instability rather than quantify vasopressor intensity.
- Renal replacement therapy (RRT): ICD-10-PCS 5A1D70Z/5A1D80Z or CPT 90935/90937/90945/90947.
- ICU length of stay (days).
- Hospital length of stay (days).
- 90-day all-cause hospital readmission.

### 2.5. Statistical Analysis

Baseline characteristics were compared using chi-square tests for categorical variables and Student’s *t*-tests or Wilcoxon rank-sum tests for continuous variables, as appropriate. To account for baseline differences between cohorts, we performed 1:1 propensity score matching (PSM) using the nearest-neighbor algorithm without replacement with a caliper width of 0.01 standard deviations of the logit of the propensity score. All 18 covariates listed above were included in the propensity score model. Covariate balance after matching was assessed using standardized mean differences (SMD), with values <0.10 considered indicative of adequate balance.

For each binary outcome, we calculated relative risks (RR) and risk differences (RD) with 95% confidence intervals (CI) at predefined follow-up intervals of 7, 30, and 90 days. For continuous outcomes, mean differences with 95% CI were calculated. To address time-to-event relationships, we performed Cox proportional hazards regression analysis in the propensity-matched cohort without additional covariate adjustment, as all measured baseline covariates were well balanced after matching. Hazard ratios (HR) with 95% CI were calculated for all primary and secondary outcomes. The proportional hazards assumption was assessed using Schoenfeld residuals.

Sensitivity and subgroup analyses included:

1. Restricted analysis: Excluding recipients transplanted from the ICU or requiring pre-transplant mechanical ventilation or vasopressors, with new PSM performed within this subgroup.
2. Restrictive frailty-definition sensitivity analysis: To improve specificity and address potential misclassification from single-code ascertainment, we repeated the primary analysis using a more restrictive, administratively defined frailty phenotype requiring at least two qualifying frailty-related ICD-10-CM codes within 12 months before transplantation. New PSM was performed within this sensitivity cohort.
3. MELD 3.0 sensitivity analysis: To assess whether findings were robust to the use of a contemporary liver disease severity metric, we repeated PSM after substituting MELD 3.0 for MELD-Na in the propensity score model.
4. Etiology-stratified analysis: Outcomes were examined within subgroups defined by primary liver disease etiology, including alcohol-associated liver disease, MASLD, viral hepatitis, and autoimmune/cholestatic liver disease, to assess consistency of frailty-outcome associations. These subgroup analyses were considered exploratory and were not powered for definitive subgroup-specific conclusions. Interaction *p*-values were used to assess statistical heterogeneity across etiologic subgroups.

Statistical significance was defined as a two-sided *p*-value <0.05. All analyses were conducted within the TriNetX analytics platform.

## 3. Results

### 3.1. Study Population

**Figure 1:**
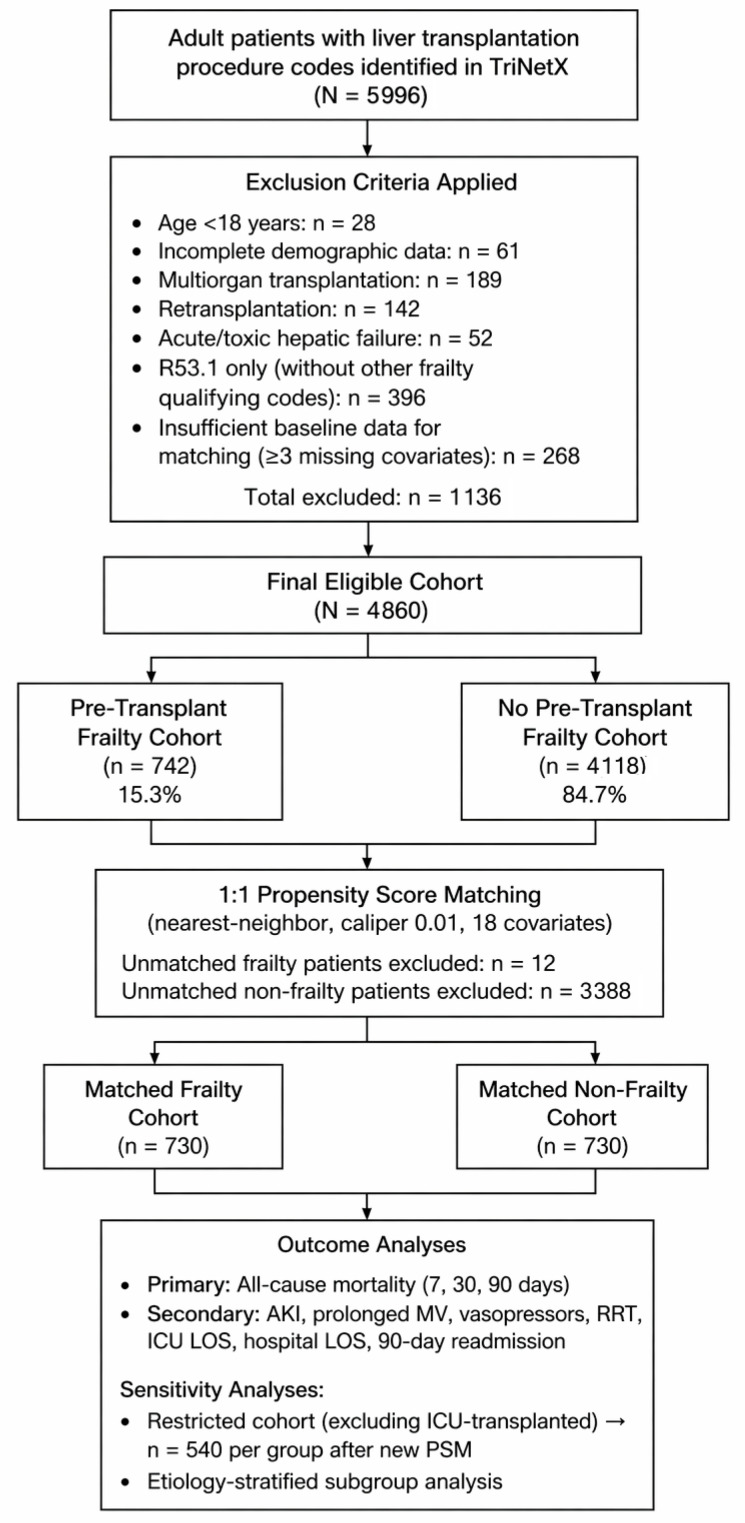
Study flow diagram showing cohort selection, exclusion criteria, propensity score matching, and outcome analyses.

A total of 5996 adult patients with liver transplantation procedure codes were identified in the TriNetX US Collaborative Research Network. After sequential application of exclusion criteria, 4860 patients comprised the final eligible cohort: 742 (15.3%) with administratively defined pre-transplant frailty and 4118 (84.7%) without. After 1:1 PSM, 730 patients remained in each group. The patient selection process is illustrated in Figure 1.

### 3.2. Baseline Characteristics

Before PSM, the administratively defined frailty cohort was significantly older, more likely to be female, and had higher prevalences of type 2 diabetes, hypertension, CKD, hepatic encephalopathy, ascites, and pre-transplant dialysis, as well as higher MELD-Na scores and worse laboratory parameters. After PSM, all 18 covariates were well balanced (all SMD < 0.10) (Table 1).

### 3.3. Primary and Secondary Outcomes

At 7 days, all-cause mortality was significantly higher in the administratively defined frailty cohort (RR 2.571; 95% CI: 1.102–5.998; *p* = 0.021). This association persisted at 30 days (RR 1.958; 95% CI: 1.224–3.133; *p* = 0.004) and 90 days (RR 1.646; 95% CI: 1.182–2.293; *p* = 0.003). Frailty was significantly associated with increased risk of AKI, prolonged mechanical ventilation, vasopressor requirement, and RRT at all assessed time points, with numerically larger relative risks at earlier postoperative time points. Ninety-day readmission was also significantly higher in frail recipients (Table 2).

### 3.4. Length of Stay and Continuous Outcomes

Frailty was associated with significantly longer hospital and ICU stays, higher peak creatinine, greater creatinine rise from baseline, and longer ventilator duration among ventilated patients (Table 3).

### 3.5. Cox Proportional Hazards Model

Cox regression confirmed significantly higher hazards for all primary and secondary outcomes in frail recipients (Table 4). The proportional hazards assumption was met for all models (Schoenfeld residual global test *p* > 0.05 for all outcomes).

### 3.6. Sensitivity Analyses

#### 3.6.1. Restricted Acuity Analysis

To address residual confounding by pre-transplant illness acuity, analyses were repeated in a restricted subgroup excluding recipients transplanted from the ICU or requiring pre-transplant mechanical ventilation or vasopressors. After a new PSM within this subgroup (*n* = 540 per group), all covariates remained balanced (all SMD < 0.10). Associations persisted but were attenuated compared with the full cohort, suggesting that administratively defined frailty was not solely a surrogate for extreme pre-transplant illness acuity (Table 5).

#### 3.6.2. Restrictive Frailty-Definition Sensitivity Analysis

To improve specificity and address potential misclassification from single-code ascertainment, the primary analysis was repeated using a more restrictive administratively defined frailty phenotype requiring at least two qualifying frailty-related ICD-10-CM codes within 12 months before transplantation. After repeated PSM, associations remained directionally consistent with the primary analysis across mortality, AKI, prolonged mechanical ventilation, vasopressor requirement, RRT, and 90-day readmission (Table 6).

#### 3.6.3. MELD 3.0 Sensitivity Analysis

To evaluate whether findings were robust to the use of a contemporary liver disease severity metric, PSM was repeated after substituting MELD 3.0 for MELD-Na in the propensity score model. The direction and magnitude of associations remained broadly consistent with the primary analysis, including for mortality, AKI, prolonged mechanical ventilation, vasopressor requirement, RRT, and 90-day readmission (Table 7).

### 3.7. Etiology-Stratified Subgroup Analysis

To explore whether associations between administratively defined frailty and early outcomes differed by underlying liver disease etiology, 30-day mortality, 7-day AKI, and 7-day prolonged mechanical ventilation were examined within etiology-defined subgroups from the matched cohort. These analyses were exploratory and were not powered for definitive subgroup-specific conclusions. Associations were directionally consistent across etiologic subgroups, although several subgroup-specific estimates did not reach statistical significance. Interaction *p*-values were non-significant, indicating no statistically detectable heterogeneity across etiologies. Therefore, these findings should be interpreted as supporting the overall consistency of the association rather than demonstrating clinically meaningful etiology-specific differences (Table 8).

## 4. Discussion

In this large propensity-matched multicenter cohort of 1460 liver transplant recipients, administratively defined pre-transplant frailty was associated with worse early post-transplant outcomes across multiple domains, including mortality, AKI, prolonged mechanical ventilation, vasopressor requirement, RRT, prolonged hospitalization, and 90-day readmission. Relative risks were numerically larger in the immediate postoperative period and smaller at later time points.

The mortality findings are directionally consistent with the landmark FrAILT study, which reported an adjusted HR of 2.13 (95% CI: 1.39–3.26) for post-transplant mortality in frail recipients defined by LFI ≥ 4.5 [5]. The present study’s 90-day mortality HR of 1.71 (95% CI: 1.28–2.28) is modestly lower, which may reflect differences in frailty ascertainment (EHR-based coding vs. validated performance-based instrument) and the resulting misclassification bias, which would be expected to attenuate effect estimates toward the null. This study builds on the FrAILT findings by showing associations between frailty and more granular early outcomes—AKI, RRT, prolonged mechanical ventilation, and hemodynamic instability—that were not reported in the FrAILT cohort [5]. These outcomes may represent potential targets for future perioperative optimization studies.

A key distinction between the present study and prior prospective frailty studies is that frailty was identified using ICD-10-CM codes rather than direct performance-based testing. Therefore, the exposure in this study should be interpreted as an administratively defined frailty phenotype or claims-based frailty phenotype, not as directly measured physiologic frailty. The included codes likely capture a heterogeneous clinical construct overlapping with frailty, sarcopenia, cachexia, malnutrition, generalized weakness, debility, disability, and mobility limitation. This approach may have lower sensitivity than prospective LFI-based assessment and may preferentially identify patients with clinically apparent functional impairment. Conversely, some included codes may reflect advanced liver disease or disability rather than frailty itself. These considerations may introduce exposure misclassification and should be considered when interpreting the findings. Prior studies have shown that claims-based frailty measures can stratify risk in administrative datasets, but they are not interchangeable with direct performance-based frailty instruments.

The association between frailty/sarcopenia and AKI is consistent with findings from a nationwide analysis of over 170,000 liver transplant recipients, which demonstrated that sarcopenia was independently associated with AKI (aOR 1.4; 95% CI: 1.32–1.49), shock (aOR 2.18), and in-hospital mortality (aOR 2.16) [13]. Similarly, frailty has been identified as an independent predictor of AKI in other surgical populations, including cardiac surgery and emergency laparotomy [14,15]. The recently published LTCI incorporated frailty alongside coronary artery disease, HCC, renal dysfunction, and diabetes into a composite index predicting 3-year post-transplant mortality [6]. The present study complements the LTCI framework by showing that administratively defined frailty was associated with early outcomes even after propensity score matching. Other recipient risk stratification models have similarly identified overlapping comorbidity domains (age, diabetes, renal dysfunction, ventilator status) as predictors of post-transplant mortality, but none have incorporated frailty as a distinct variable or examined its association with granular early postoperative outcomes [16,17].

An important observation was that relative risks were numerically larger at earlier postoperative time points and smaller at later time points across several measured outcomes. This may suggest that administratively defined pre-transplant frailty is most apparent as a risk marker during the immediate physiologic stress of transplantation, when cardiopulmonary reserve, respiratory muscle strength, renal vulnerability, and nutritional reserve may be challenged [18,19]. However, this temporal pattern should be interpreted cautiously. Alternative explanations include survivor bias, as the sickest, frail recipients who experience early mortality are no longer in the risk set at later time points; competing risks, as death may preclude the occurrence or capture of non-fatal outcomes; and differential discharge or follow-up patterns, particularly in a federated EHR database where post-discharge care outside participating healthcare organizations may not be fully captured [10,11]. Because formal time-varying effect modeling was not performed, these findings should be considered descriptive and hypothesis-generating rather than definitive evidence of biological attenuation of frailty-associated risk. These observations may help identify early postoperative outcomes for future studies of perioperative optimization, including early mobilization, nephroprotective strategies, and ventilator-weaning protocols, but whether such interventions modify risk in frail recipients requires prospective evaluation [20,21,22].

The etiology-stratified analysis was exploratory. Although point estimates varied numerically across liver disease etiologies, subgroup comparisons were underpowered for independent significance, and interaction *p*-values were non-significant. Therefore, there was no statistically detectable heterogeneity in the association between administratively defined frailty and early outcomes across etiologies. These findings should be interpreted as supporting the overall consistency of the association rather than suggesting meaningful etiology-specific differences.

The persistence of associations in the restricted cohort excluding ICU-transplant recipients and those requiring pre-transplant mechanical ventilation or vasopressors supports the robustness of the observed findings. However, this analysis should be interpreted cautiously because residual confounding by unmeasured illness acuity and transplant-specific factors cannot be excluded. The attenuation of effect sizes in the restricted cohort may reflect exclusion of the highest-acuity recipients rather than proving that administratively defined frailty represents a distinct biologic vulnerability state.

Taken together, these findings may have several clinical implications. First, they support further evaluation of structured frailty assessment during pre-transplant evaluation, consistent with the 2025 AASLD/AST Practice Guideline recommendation for LFI measurement [4]. Second, the association of administratively defined frailty with AKI, prolonged mechanical ventilation, and hemodynamic instability may help identify early postoperative outcomes that warrant closer monitoring and future study within perioperative optimization pathways, including nephroprotective strategies, respiratory support planning, early mobilization, and ICU care protocols [20,23]. Third, although the observed temporal pattern should be interpreted cautiously, these findings suggest that the immediate postoperative period may be a particularly important window for studying targeted interventions in vulnerable recipients. This aligns with emerging prehabilitation literature showing that frailty and functional capacity may be modifiable before transplantation, including improvements in LFI, 6 min walk test performance, VO2, and Short Physical Performance Battery measures in selected transplant candidates [8,9,24]. Fourth, the directionally consistent findings across etiologic subgroups suggest that frailty assessment may be relevant across liver disease etiologies, although subgroup analyses were exploratory and not powered for definitive etiology-specific conclusions. Finally, prior evidence that liver transplantation provides survival benefit across frailty strata reinforces that frailty-related risk information should be used to guide optimization and perioperative planning rather than to exclude candidates from transplantation [4,7].

These implications are strengthened by several features of the present study. The TriNetX platform provided a large, geographically diverse, multicenter cohort that is broadly representative of US transplant centers [10,11]. The sample size (730 matched pairs) exceeded the total FrAILT cohort (1166 in total) and enabled assessment of multiple secondary outcomes with adequate statistical power [5]. The comprehensive propensity matching on 18 covariates provided robust confounding adjustment. The multi-time point outcome assessment (7, 30, 90 days) enabled characterization of temporal attenuation patterns. The restricted acuity, restrictive frailty-definition, and MELD 3.0 sensitivity analyses support the robustness of the findings, while the etiology-stratified analysis provides exploratory evidence of directional consistency across liver disease etiologies.

Despite these strengths, several limitations warrant consideration. First, frailty was defined using ICD-10-CM administrative codes rather than validated performance-based instruments such as the LFI [2,3]. Therefore, the exposure should be interpreted as administratively defined frailty rather than directly measured physiologic frailty. Administrative frailty coding likely underestimates true frailty prevalence (15.3% in this study vs. 20–33% in prospective studies using LFI) and may introduce misclassification bias [5,6]. Although such misclassification would generally be expected to bias estimates toward the null, the included codes may also capture overlapping domains of sarcopenia, cachexia, malnutrition, weakness, debility, disability, mobility limitation, and advanced liver disease severity. Sensitivity analysis requiring at least two qualifying frailty-related ICD-10-CM codes yielded directionally consistent findings. Second, TriNetX does not capture several transplant-specific variables that may influence early postoperative outcomes, including donor age, donor type (DCD vs. DBD), graft quality, cold ischemia time, warm ischemia time, intraoperative blood loss, transfusion requirement, surgical complexity, center-level volume, and center-specific perioperative practices [16]. These factors may influence AKI, ICU length of stay, mechanical ventilation duration, vasopressor requirement, and early mortality through ischemia–reperfusion injury, hemodynamic instability, transfusion exposure, operative complexity, and variation in institutional care pathways. Therefore, residual confounding by unmeasured donor, intraoperative, graft-related, and center-level factors cannot be excluded. Third, several outcomes were identified using structured EHR and administrative data rather than manual chart adjudication. AKI was defined using ICD-10-CM codes rather than KDIGO creatinine- or urine output-based criteria [12]. This likely underestimates mild AKI and preferentially captures clinically recognized moderate-to-severe AKI. Vasopressor requirement may be underestimated if medication administration records are incomplete or overestimated if vasopressors were administered briefly for transient perioperative hypotension. Mechanical ventilation duration may be imprecisely captured because ventilator flow-sheet data and exact extubation timing are not uniformly available. Because these outcome definitions were applied uniformly to both cohorts, misclassification is likely to be largely nondifferential, although the exact direction and magnitude of bias cannot be determined. Fourth, the federated EHR design may result in incomplete follow-up if patients receive post-transplant care at non-participating institutions [10,11]. This limitation is particularly relevant for post-discharge outcomes such as readmission and later time point events. Fifth, MELD-Na rather than MELD 3.0 was retained as the primary composite liver disease severity variable to maintain comparability with prior transplant outcomes and frailty literature [5,6,16,17]. However, MELD 3.0 has been adopted for contemporary allocation and may better reflect current liver disease severity assessment [25]. In this study, sensitivity analysis substituting MELD 3.0 for MELD-Na in the propensity score model yielded directionally consistent findings. Sixth, the observed pattern of numerically smaller relative risks at later time points was descriptive. Formal time-varying effect modeling was not performed, and this pattern may reflect survivor bias, competing risks, differential discharge patterns, or incomplete capture of post-discharge outcomes rather than true biological attenuation of frailty-associated risk. Finally, the etiology-stratified analyses were exploratory and underpowered for definitive subgroup comparisons. Non-significant interaction *p*-values indicate no statistically detectable heterogeneity across etiologies. Given the observational design and administrative data source, all findings should be interpreted as associative rather than causal.

## 5. Conclusions

In this large multicenter propensity-matched cohort, administratively defined pre-transplant frailty was associated with worse early outcomes after liver transplantation, including higher mortality, AKI, prolonged mechanical ventilation, RRT, hemodynamic instability, longer hospitalization, and 90-day readmission. Relative risks were numerically larger in the immediate postoperative period and smaller at later time points, although this temporal pattern requires confirmation using formal time-varying analyses. Associations were directionally consistent across liver disease etiologies, with no statistically significant evidence of heterogeneity. Sensitivity analyses using a more restrictive frailty definition and substituting MELD 3.0 for MELD-Na yielded consistent findings. Because frailty and postoperative outcomes were identified using structured EHR and administrative data, these findings should be interpreted as associative and hypothesis-generating. Prospective studies using validated frailty instruments and granular donor, intraoperative, graft-related, and center-level variables are needed to confirm these findings and evaluate whether targeted prehabilitation or perioperative optimization can modify early post-transplant risk.

## Figures and Tables

**Table 1:** Baseline demographic and clinical characteristics before and after propensity score matching.

Characteristic	Before PSM Frailty (N = 742)	Before PSM No Frailty (N = 4118)	*p* Value	SMD	After PSM Frailty (N = 730)	After PSM No Frailty (N = 730)	*p* Value	SMD
Age, years	59.8 ± 10.7	56.4 ± 11.3	<0.001	0.307	59.7 ± 10.7	59.5 ± 10.9	0.742	0.018
Female	286 (38.5%)	1344 (32.6%)	0.002	0.124	281 (38.5%)	276 (37.8%)	0.791	0.014
White	501 (67.5%)	2751 (66.8%)	0.718	0.015	494 (67.7%)	489 (67.0%)	0.779	0.015
Black	108 (14.6%)	587 (14.3%)	0.834	0.009	106 (14.5%)	102 (14.0%)	0.786	0.016
Hispanic	92 (12.4%)	536 (13.0%)	0.656	0.018	90 (12.3%)	95 (13.0%)	0.694	0.022
Type 2 diabetes	311 (41.9%)	1325 (32.2%)	<0.001	0.202	304 (41.6%)	298 (40.8%)	0.760	0.016
Hypertension	422 (56.9%)	2077 (50.4%)	0.001	0.131	416 (57.0%)	410 (56.2%)	0.770	0.016
CKD	247 (33.3%)	1039 (25.2%)	<0.001	0.179	242 (33.2%)	236 (32.3%)	0.714	0.018
Hepatic encephalopathy	388 (52.3%)	1729 (42.0%)	<0.001	0.209	381 (52.2%)	374 (51.2%)	0.698	0.020
Ascites	471 (63.5%)	2241 (54.4%)	<0.001	0.187	463 (63.4%)	455 (62.3%)	0.677	0.023
Hepatocellular carcinoma	141 (19.0%)	896 (21.8%)	0.091	0.069	140 (19.2%)	144 (19.7%)	0.821	0.013
Pre-transplant dialysis	86 (11.6%)	307 (7.5%)	<0.001	0.141	82 (11.2%)	79 (10.8%)	0.810	0.013
MELD-Na	24.8 ± 7.3	22.6 ± 6.8	<0.001	0.312	24.7 ± 7.2	24.5 ± 7.1	0.645	0.028
MELD 3.0	25.6 ± 7.5	23.1 ± 7.0	<0.001	0.345	25.4 ± 7.4	25.2 ± 7.3	0.604	0.027
Creatinine, mg/dL	1.42 ± 0.72	1.26 ± 0.65	<0.001	0.232	1.41 ± 0.71	1.39 ± 0.69	0.650	0.029
Bilirubin, mg/dL	7.6 ± 6.3	6.5 ± 5.7	<0.001	0.184	7.5 ± 6.2	7.4 ± 6.0	0.820	0.016
INR	1.91 ± 0.58	1.78 ± 0.52	<0.001	0.235	1.90 ± 0.58	1.89 ± 0.56	0.761	0.018
Sodium, mmol/L	131.2 ± 5.7	132.5 ± 5.3	<0.001	0.237	131.3 ± 5.6	131.5 ± 5.4	0.528	0.036
Albumin, g/dL	2.68 ± 0.49	2.83 ± 0.46	<0.001	0.316	2.69 ± 0.49	2.70 ± 0.47	0.711	0.021

Abbreviations: CKD = chronic kidney disease; INR = international normalized ratio; MELD-Na = Model for End-Stage Liver Disease–Sodium; MELD 3.0 = Model for End-Stage Liver Disease 3.0; PSM = propensity score matching; SMD = standardized mean difference. Continuous variables are presented as mean ± standard deviation and categorical variables as *n* (%).

**Table 2:** Relative risks and risk differences for clinical outcomes after propensity score matching.

Outcome	Time Point	Frailty (*n*/N)	No Frailty (*n*/N)	RD (95% CI)	RR (95% CI)	*p* Value
All-cause mortality	7 Days	18/730	7/730	0.015 (0.002, 0.029)	2.571 (1.102–5.998)	0.021
30 Days	47/730	24/730	0.032 (0.010, 0.055)	1.958 (1.224–3.133)	0.004	
90 Days	79/730	48/730	0.042 (0.014, 0.070)	1.646 (1.182–2.293)	0.003	
Acute kidney injury	7 Days	219/730	154/730	0.089 (0.043, 0.135)	1.422 (1.196–1.692)	<0.001
30 Days	290/730	224/730	0.090 (0.041, 0.140)	1.295 (1.132–1.481)	<0.001	
90 Days	331/730	275/730	0.076 (0.026, 0.126)	1.204 (1.083–1.340)	0.001	
Prolonged mechanical ventilation	7 Days	196/730	121/730	0.103 (0.060, 0.146)	1.620 (1.334–1.967)	<0.001
30 Days	218/730	147/730	0.097 (0.051, 0.143)	1.483 (1.240–1.774)	<0.001	
Vasopressor requirement/hemodynamic instability	7 Days	241/730	162/730	0.108 (0.062, 0.154)	1.488 (1.251–1.771)	<0.001
30 Days	260/730	190/730	0.096 (0.048, 0.145)	1.368 (1.173–1.595)	<0.001	
Renal replacement therapy	7 Days	51/730	29/730	0.030 (0.008, 0.053)	1.759 (1.127–2.747)	0.012
30 Days	74/730	49/730	0.034 (0.008, 0.060)	1.510 (1.056–2.159)	0.022	
90-day readmission	90 Days	167/730	128/730	0.053 (0.014, 0.092)	1.305 (1.073–1.587)	0.007

Abbreviations: CI = confidence interval; RD = risk difference; RR = relative risk.

**Table 3:** Length of stay and selected continuous outcomes after propensity score matching.

Outcome	Frailty (N = 730)	No Frailty (N = 730)	Mean Difference (95% CI)	*p* Value
Hospital length of stay, days	18.6 ± 9.7	14.9 ± 8.3	3.7 (2.8 to 4.6)	<0.001
ICU length of stay, days	7.3 ± 4.8	5.9 ± 4.1	1.4 (0.9 to 1.9)	<0.001
Peak creatinine during admission, mg/dL	2.10 ± 1.02	1.80 ± 0.91	0.30 (0.20 to 0.40)	<0.001
Change in creatinine from baseline to peak, mg/dL	+0.68 ± 0.74	+0.41 ± 0.63	0.27 (0.20 to 0.34)	<0.001
Ventilator days among ventilated patients	5.8 ± 4.3	4.1 ± 3.6	1.7 (1.0 to 2.4)	<0.001

Abbreviations: CI = confidence interval; ICU = intensive care unit. Continuous variables are presented as mean ± standard deviation.

**Table 4:** Cox proportional hazards models for outcomes associated with administratively defined pre-transplant frailty in the propensity-matched cohort.

Outcome	HR	Coefficient	SE	z	*p* Value	95% CI
All-cause mortality	1.71	0.536	0.148	3.62	<0.001	1.28–2.28
Acute kidney injury	1.38	0.322	0.067	4.81	<0.001	1.21–1.57
Prolonged mechanical ventilation	1.59	0.464	0.081	5.73	<0.001	1.36–1.86
Vasopressor requirement/hemodynamic instability	1.44	0.365	0.074	4.93	<0.001	1.24–1.67
Renal replacement therapy	1.47	0.385	0.139	2.77	0.006	1.12–1.94
90-day readmission	1.31	0.270	0.088	3.07	0.002	1.10–1.56

Abbreviations: CI = confidence interval; HR = hazard ratio; SE = standard error.

**Table 5:** Restricted subgroup analysis excluding recipients transplanted from the ICU or requiring pre-transplant mechanical ventilation or vasopressors (post-PSM, *n* = 540 per group).

Outcome	Time Point	Events Frailty	Events No Frailty	RR (95% CI)	HR (95% CI)	*p* Value
All-cause mortality	30 Days	29	17	1.74 (1.01–2.99)	1.52 (1.08–2.15)	0.017
AKI	7 Days	141	108	1.31 (1.08–1.59)	1.27 (1.08–1.49)	0.003
Prolonged mechanical ventilation	7 Days	126	92	1.37 (1.09–1.73)	1.34 (1.10–1.63)	0.004
Vasopressor requirement	7 Days	151	117	1.29 (1.07–1.57)	1.26 (1.05–1.51)	0.010

Abbreviations: AKI = acute kidney injury; CI = confidence interval; HR = hazard ratio; ICU = intensive care unit; PSM = propensity score matching; RR = relative risk.

**Table 6:** Sensitivity analysis using a restrictive administratively defined frailty phenotype requiring ≥2 qualifying frailty-related ICD-10-CM codes within 12 months before liver transplantation.

Outcome	Time Point	Frailty *n*/N	No Frailty *n*/N	RD, %	RR	95% CI	*p* Value
All-cause mortality	7 days	14/512	6/512	1.6	2.33	0.90–6.03	0.071
All-cause mortality	30 days	36/512	20/512	3.1	1.80	1.06–3.05	0.027
All-cause mortality	90 days	61/512	39/512	4.3	1.56	1.06–2.30	0.021
AKI	7 days	158/512	116/512	8.2	1.36	1.12–1.66	0.002
AKI	30 days	204/512	163/512	8.0	1.25	1.07–1.46	0.005
AKI	90 days	236/512	202/512	6.6	1.17	1.01–1.35	0.034
Prolonged mechanical ventilation	7 days	143/512	96/512	9.2	1.49	1.19–1.87	<0.001
Vasopressor requirement/hemodynamic instability	7 days	169/512	122/512	9.2	1.39	1.14–1.68	0.001
RRT	7 days	38/512	24/512	2.7	1.58	0.96–2.59	0.068
90-day readmission	90 days	118/512	92/512	5.1	1.28	1.01–1.62	0.039

Abbreviations: AKI = acute kidney injury; CI = confidence interval; ICD-10-CM = International Classification of Diseases, Tenth Revision, Clinical Modification; RD = risk difference; RR = relative risk; RRT = renal replacement therapy.

**Table 7:** Sensitivity analysis using MELD 3.0 instead of MELD-Na in the propensity score matching model.

Outcome	Time Point	Frailty *n*/N	No Frailty *n*/N	RD, %	RR	95% CI	*p* Value
All-cause mortality	7 days	17/718	7/718	1.4	2.43	1.02–5.78	0.038
All-cause mortality	30 days	45/718	24/718	2.9	1.88	1.17–3.02	0.008
All-cause mortality	90 days	76/718	49/718	3.8	1.55	1.10–2.18	0.010
AKI	7 days	214/718	153/718	8.5	1.40	1.17–1.67	<0.001
AKI	30 days	283/718	222/718	8.5	1.27	1.11–1.46	<0.001
AKI	90 days	324/718	273/718	7.1	1.19	1.06–1.33	0.003
Prolonged mechanical ventilation	7 days	191/718	122/718	9.6	1.57	1.29–1.91	<0.001
Prolonged mechanical ventilation	30 days	212/718	148/718	8.9	1.43	1.20–1.71	<0.001
Vasopressor requirement/hemodynamic instability	7 days	236/718	164/718	10.0	1.44	1.21–1.71	<0.001
Vasopressor requirement/hemodynamic instability	30 days	254/718	192/718	8.6	1.32	1.13–1.54	<0.001
RRT	7 days	49/718	30/718	2.6	1.63	1.04–2.56	0.031
RRT	30 days	71/718	51/718	2.8	1.39	0.98–1.98	0.063
90-day readmission	90 days	164/718	129/718	4.9	1.27	1.04–1.55	0.017

Abbreviations: AKI = acute kidney injury; CI = confidence interval; MELD = Model for End-Stage Liver Disease; RD = risk difference; RR = relative risk; RRT = renal replacement therapy.

**Table 8:** Etiology-Stratified Subgroup Analysis: 30-Day Mortality, 7-Day AKI, and 7-Day Prolonged Mechanical Ventilation by Liver Disease Etiology (Matched Cohort).

Etiology	*n* (F/NF)	30-Day Mortality Events (F vs. NF)	30-Day Mortality RR (95% CI)	30-Day Mortality *p*	7-Day AKI Events (F vs. NF)	7-Day AKI RR (95% CI)	7-Day AKI *p*	7-Day MV Events (F vs. NF)	7-Day MV RR (95% CI)	7-Day MV *p*
Alcohol-associated	263/259	18 vs. 9	1.97 (0.90–4.31)	0.084	86 vs. 54	1.57 (1.17–2.11)	0.003	74 vs. 43	1.70 (1.22–2.36)	0.002
MASLD	117/114	10 vs. 4	2.44 (0.79–7.53)	0.108	38 vs. 24	1.54 (0.98–2.43)	0.058	30 vs. 18	1.62 (0.96–2.75)	0.068
Viral hepatitis	175/179	12 vs. 7	1.75 (0.71–4.33)	0.218	58 vs. 44	1.35 (0.97–1.88)	0.076	51 vs. 34	1.53 (1.05–2.24)	0.026
Autoimmune/cholestatic	81/84	4 vs. 2	2.07 (0.39–11.0)	0.389	21 vs. 17	1.28 (0.73–2.25)	0.387	22 vs. 14	1.63 (0.89–2.98)	0.109
Other/cryptogenic	94/94	3 vs. 2	1.50 (0.26–8.76)	0.651	16 vs. 15	1.07 (0.56–2.03)	0.844	19 vs. 12	1.58 (0.82–3.07)	0.170
Total (matched cohort)	730/730	47 vs. 24	1.96 (1.22–3.13)	0.004	219 vs. 154	1.42 (1.20–1.69)	0.001	196 vs. 121	1.62 (1.33–1.97)	0.001
Interaction *p* value				0.872		0.641				0.914

Abbreviations: AKI = acute kidney injury; CI = confidence interval; F = frailty; MASLD = metabolic dysfunction-associated steatotic liver disease; MV = mechanical ventilation; NF = no frailty; RR = relative risk. Interaction *p*-values test for heterogeneity of the frailty effect across etiologic subgroups using the Breslow-Day test. Non-significant interaction *p*-values indicate that the frailty-outcome association was consistent across etiologies. Individual subgroups were underpowered for independent significance in several strata.

## Data Availability

The data that support the findings of this study are available from TriNetX, but restrictions apply to the availability of these data, which were used under license for the current study and are therefore not publicly available. Data may be available from the authors upon reasonable request and with permission of TriNetX.

## Abbreviations

The following abbreviations are used in this manuscript:

AASLD	American Association for the Study of Liver Diseases
AKI	Acute kidney injury
AST	American Society of Transplantation
CI	Confidence interval
CKD	Chronic kidney disease
CPT	Current Procedural Terminology
DBD	Donation after brain death
DCD	Donation after circulatory death
EHR	Electronic health record
HCC	Hepatocellular carcinoma
HCO	Healthcare organization
HIPAA	Health Insurance Portability and Accountability Act
HR	Hazard ratio
ICD-10-CM	International Classification of Diseases, Tenth Revision, Clinical Modification
ICD-10-PCS	International Classification of Diseases, Tenth Revision, Procedure Coding System
ICU	Intensive care unit
INR	International normalized ratio
KDIGO	Kidney Disease: Improving Global Outcomes
LFI	Liver Frailty Index
LOS	Length of stay
LTCI	Liver Transplant Comorbidity Index
MASLD	Metabolic dysfunction-associated steatotic liver disease
MELD 3.0	Model for End-Stage Liver Disease 3.0
MELD-Na	Model for End-Stage Liver Disease–Sodium
OPTN	Organ Procurement and Transplantation Network
PSM	Propensity score matching
RD	Risk difference
RR	Relative risk
RRT	Renal replacement therapy
SMD	Standardized mean difference
STROBE	Strengthening the Reporting of Observational Studies in Epidemiology
